# OnEX: Exploring changes in life science ontologies

**DOI:** 10.1186/1471-2105-10-250

**Published:** 2009-08-13

**Authors:** Michael Hartung, Toralf Kirsten, Anika Gross, Erhard Rahm

**Affiliations:** 1 Interdisciplinary Centre for Bioinformatics, University of Leipzig, Härtelstraße 16-18, 04107 Leipzig, Germany; 2 Institute for Medical Informatics, Statistics and Epidemiology, University of Leipzig, Härtelstraße 16-18, 04107 Leipzig, Germany; 3 Department of Computer Science, University of Leipzig, PO box 100920, 04009 Leipzig, Germany

## Abstract

**Background:**

Numerous ontologies have recently been developed in life sciences to support a consistent annotation of biological objects, such as genes or proteins. These ontologies underlie continuous changes which can impact existing annotations. Therefore, it is valuable for users of ontologies to study the stability of ontologies and to see how many and what kind of ontology changes occurred.

**Results:**

We present **OnEX **(Ontology Evolution EXplorer) a system for exploring ontology changes. Currently, **OnEX **provides access to about 560 versions of 16 well-known life science ontologies. The system is based on a three-tier architecture including an ontology version repository, a middleware component and the **OnEX **web application. Interactive workflows allow a systematic and explorative change analysis of ontologies and their concepts as well as the semi-automatic migration of out-dated annotations to the current version of an ontology.

**Conclusion:**

**OnEX **provides a user-friendly web interface to explore information about changes in current life science ontologies. It is available at .

## Background

### Life science ontologies

Ontologies have become increasingly important in life sciences [1,2]. They consist of a set of concepts denoted by terms describing and structuring a domain of interest. Concepts are interconnected by different relationship types such as is_a and part_of relationships. A heavily used ontology is the Gene Ontology (GO) [3] providing sub-ontologies for molecular functions (MF), biological processes (BP) and cellular components (CC). A wide range of life science ontologies is made available by the OBO (Open Biomedical Ontologies) Foundry [4]. The ontologies cover various life science disciplines, such as anatomy, health, biochemistry or phenotype. Other biomedical ontologies consider clinical and disease-related issues (for instance the NCI Thesaurus [5], SNOMED CT [6] or OMIM [7]). Due to their different focus and usage the developed ontologies vary in their size and complexity. For example, some OBO ontologies consist of only a few hundred concepts while others, such as the GO possess up to several ten thousand concepts.

There are different kinds of applications of life science ontologies. They are used for the annotation of biological objects, such as gene products and proteins. Particularly, biological objects are associated ("annotated") with ontology concepts to consistently and semantically describe their properties, for example the molecular functions and biological processes in which proteins are involved. For instance, the human protein *Tubulin-specific chaperone D *[Swiss-Prot:Q9BTW9] is associated with GO concepts GO:0007025, GO:0051087, GO:0005874, thereby expressing that the protein is involved in the biological process *beta-tubulin folding *(GO:0007025), is associated with the molecular function *chaperone binding *(GO:0051087) and that it acts in the cellular component *microtubule *(GO:0005874). Such annotations can be specified manually (for example based on experimental results) or derived automatically (for example by data mining techniques). There are different data sources providing GO annotations for various species, examples are GOA [8], Swiss-Prot [9], Ensembl [10], MGD [11] or AgBase [12]. In a wide range of applications ontologies facilitate the structuring of and the focused search within large data sources. For instance, the GoPubMed application [13] makes use of MeSH [14] and GO to classify millions of articles of PubMed [15]. Users can find relevant articles significantly faster by navigating and filtering along the applied ontologies. Another ontology application is the standardization of data exchange formats in heterogeneous environments by providing a common and explicit background. For example, the caBIG project [16] utilizes the NCI Thesaurus as a foundation for defining metadata and sharing data objects in their grid environment. Metadata stored in the central caDSR repository are semantically described by referring to concepts of the Thesaurus. Hence, ontology concepts are associated to metadata compared to the more common annotation of data objects (instances).

### Ontology evolution

Usually, life science ontologies are explicitly modeled by ontology developers and scientists. The evolution of these ontologies is based on specific community agreements (at least among the ontology developers) and influenced by advances in the domain knowledge to be included in the ontologies. New research results/insights and new agreements may lead to additions or revisions of ontology elements. As a result ontologies evolve continuously and a sequence of ontology versions is provided where each version represents the state of an ontology at a specific point in time. The different versions are the basis of our change analysis. For instance, our analysis showed that in the last five years the number of concepts in GO and NCI Thesaurus more than doubled (from 13,163 to 28,250 and from 28,740 to 68,862, respectively). While most changes are additions of new concepts, many concepts have been deleted or declared obsolete (about 25 and 50 per month in GO and NCI Thesaurus, respectively). Setting an "obsolete status" for concepts is a common alternative to physically deleting a concept of an ontology. Both deleted and obsolete concepts result in a similar revision of the information represented in an ontology and may indicate a reduced stability of the ontology which negatively affects ontology usage. In particular, annotations referring to deleted or obsolete concepts are no longer valid and may have to be deleted or adapted. Other ontology changes such as additions also influence ontology usage, e.g., since they may trigger the addition of new annotations. Hence, frequent changes may impact the results of annotation analysis studies and indicate a need to rerun an analysis. Thus, there is a need to make ontology users aware of the evolution of an ontology including the quantity and quality of changes that occurred. Since not all parts of an ontology evolve at the same rate, it is useful to support the look-up of the change history for specific ontology concepts of interest, for example to see older concept names. This information may be used by curators when creating manual annotations. It would also be valuable to semi-automatically migrate out-dated annotations to newer ontology versions instead of performing a time-consuming manual adaptation.

There has been little research and support on ontology evolution and its impact for the life sciences. Several tools support change management and versioning of ontologies, e.g., the Protégé tool [17], the KAON infrastructure [18], or OBO edit [19] focusing on the OBO ontology file format. However, these approaches do not consider the impact of ontology changes on related data such as annotated biological objects or analysis results. Information on changes of current ontologies (history tracking) is primarily limited to mailing lists and reports by the ontology distributors. For instance, the GO consortium summarizes changes on the Gene Ontology in a monthly report which is available on their website . Mailing lists notify interested users about changes and modifications of OBO ontologies at . These approaches textually describe changes but cannot directly be used for automatic change processing such as an automatic migration of ontology-related data.

Theoretical aspects of ontology evolution have been studied for Semantic Web languages such as RDF [20] and OWL [21]. Stojanovic et al. formalize the process of ontology evolution [22] and propose strategies to unambiguously handle critical changes during evolution [23]. Klein investigates versioning of ontologies in a framework [24]. Noy investigates change operations describing the evolution (difference) between ontology versions [25]. Oliver et al. [26] present a concept model, typical change operations and a change-documentation model for change management of controlled medical terminologies.

There have been some studies to quantify the evolution of specific life science ontologies and terminologies. For instance, Ceusters has studied the evolution of concepts in SNOMED CT [27]. Simple change statistics such as the evolution of number of concepts, relationships and paths have been determined for the Gene Ontology [28]. In our previous work [29], we propose a generic evolution model and measures to study the evolution of ontologies, annotations and ontology-mappings. This framework serves as the basis for the **OnEX **system presented in this paper.

### Online ontology tools

There are several online tools providing interfaces to query and search life science ontologies and related data. The well-known online browsers AmiGO [30] and QuickGO [31] allow to query the Gene Ontology including ontology concepts and associated gene products. Results are presented utilizing the graph structure of GO. Users can filter results and download them for further processing. Other online portals tend to integrate multiple ontologies into a single browser that offers a common query interface for all ontologies. For instance, the EBI Ontology Lookup Service (OLS) [32] is a web-based application allowing searching and browsing of approx. 60 life science ontologies within a central portal. The Terminology Browser of the NCI [33] offers a common interface to ontologies such as the NCI Thesaurus, Gene Ontology and SNOMED CT. However, the functionality of these browsers is limited to browsing and searching in one ontology version, mostly the latest one. Historical information about former ontology versions and changes on ontology concepts are rarely provided. For example, the NCI Terminology Browser merely provides the creation and modification dates for concepts.

There exist only few online tools or web services for comparing versions of an ontology. The OWS framework presented in [34] proposes an architecture for ontology access and manipulation through web services. A diff web service takes as input two versions of an OWL ontology and returns their structural diff (concepts that were added, deleted or changed). Another web service for comparison of ontology versions is described in [35]. The service accepts a pair of ontologies written in OWL and returns a numeric value which represents their semantic difference. To the best of our knowledge GOChase [36] is the only online tool providing more detailed historical information about ontology concepts. However, the tool is specific to the Gene Ontology and is no longer maintained. Modification histories are based on GO accession numbers and the tool finds incorrect hyperlinks to GO concepts. Newer GO concepts introduced since 2006 are not captured. In contrast to GOChase, we focus on exploring and evaluating quantitative changes in many life science ontologies. Our system is not limited to one specific ontology but extensible so that further ontologies can be added in the future.

### Presenting OnEX

As mentioned in the previous sections, there is currently no online tool available for users to analyze the evolution of ontologies represented in different ontology versions. Ontology browsers do not support history tracking but at most provide creation and modification dates of concepts. To address these shortcomings we designed and implemented the **OnEX **toolkit to serve the community, especially bioinformaticians and biologists working with life science ontologies and ontology-related data.

The rest of the paper is organized as follows: The implementation section presents an overview of the **OnEX **system architecture. Next we discuss the import and versioning of ontologies within the system. The **OnEX **web application and its usage workflows are described and illustrated with sample application scenarios. The results section briefly describes the current state of the application. We finally conclude and outline possibilities for future work.

## Implementation

### Overview

**Figure 1:**
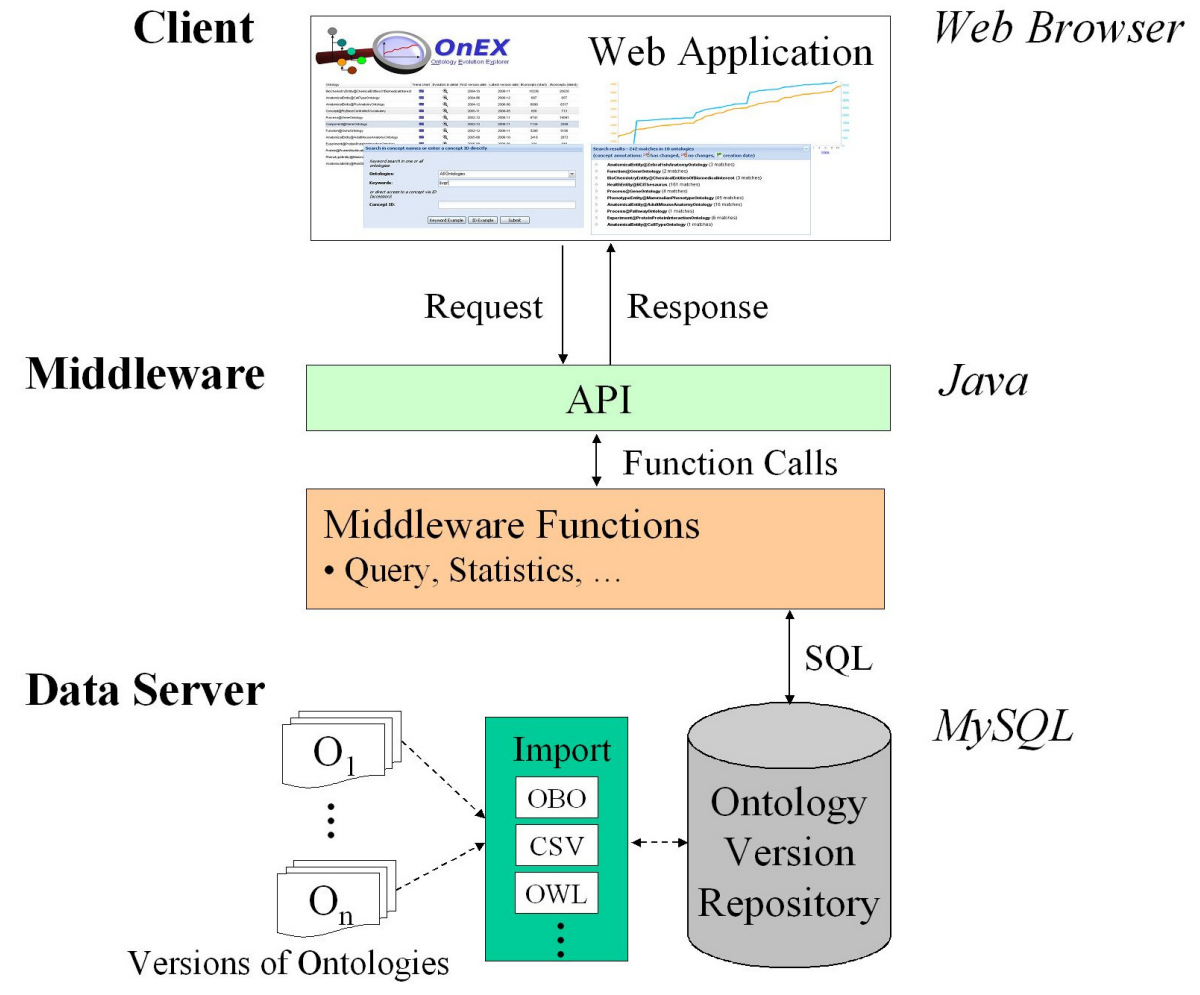
**OnEX architecture**. Three-tier architecture of the **OnEX **system.

The **OnEX **system is based on a three-tier architecture displayed in Figure 1. The backend of the system consists of a data server (MySQL database) for storage and management of numerous ontology versions and corresponding statistics. Import modules allow the integration of ontology versions in various formats. A middleware component implemented in Java provides a common API to applications or visualization components. The middleware utilizes core functions such as query methods which use SQL calls to access the ontology version repository. The web application itself is platform-independent (usable in different web browsers) and is based on the Google Web Toolkit [37] as well as the Ext GWT library [38] for building rich internet applications. The modular architecture could also support additional applications besides the online tool presented herein. In particular, web services could be added for programmatic access to the change analysis primitives as well as the ontology versions.

### Ontology import and versioning

The import function utilizes the public archives of the ontology distributors, for example the CVS repositories of the OBO ontologies or the archive of Gene Ontology. Each ontology version integrated in **OnEX **is described by a version number and a release timestamp. A new ontology version is integrated by comparing elements of the new version with the latest available version in the repository. Elements include (1) concepts, (2) relationships between concepts, and (3) attributes, such as the obsolete status or alternative synonyms of a concept.

The comparison is based on our evolution model proposed in [29] which distinguishes between the following change types:

• *add *: addition of an element

• *del *: deletion of an element

• *value change*: value of an element has changed

Change detection between two succeeding ontology versions is based on the comparison of accession numbers. An added element only exists in the new version. In contrast, a deleted element is available in the old version however not in the new version. A value change is detected if an element is available in both versions having different values, for example the name of a concept has been modified. Changes to the values of the obsolete status are especially important as they indicate whether an element has become obsolete.

The differences between two succeeding ontology versions are used to update the version repository of **OnEX**. Each element in the repository is assigned a start timestamp (date when the element was introduced) and an end timestamp describing the time period within which it is valid. An unspecified end timestamp indicates that an element is still valid. In case of an addition a new element is inserted with a start timestamp of its first occurrence. A deletion results in the adaptation of an element's end timestamp to the date of its last valid occurrence. In case of changes the old entries are assigned according end timestamps; furthermore new entries are created. Inspection of the obsolete attribute allows to detect concepts that have become obsolete. Another important change type are concept fusions which can be determined by studying alternative synonyms of concepts.

**Figure 2:**
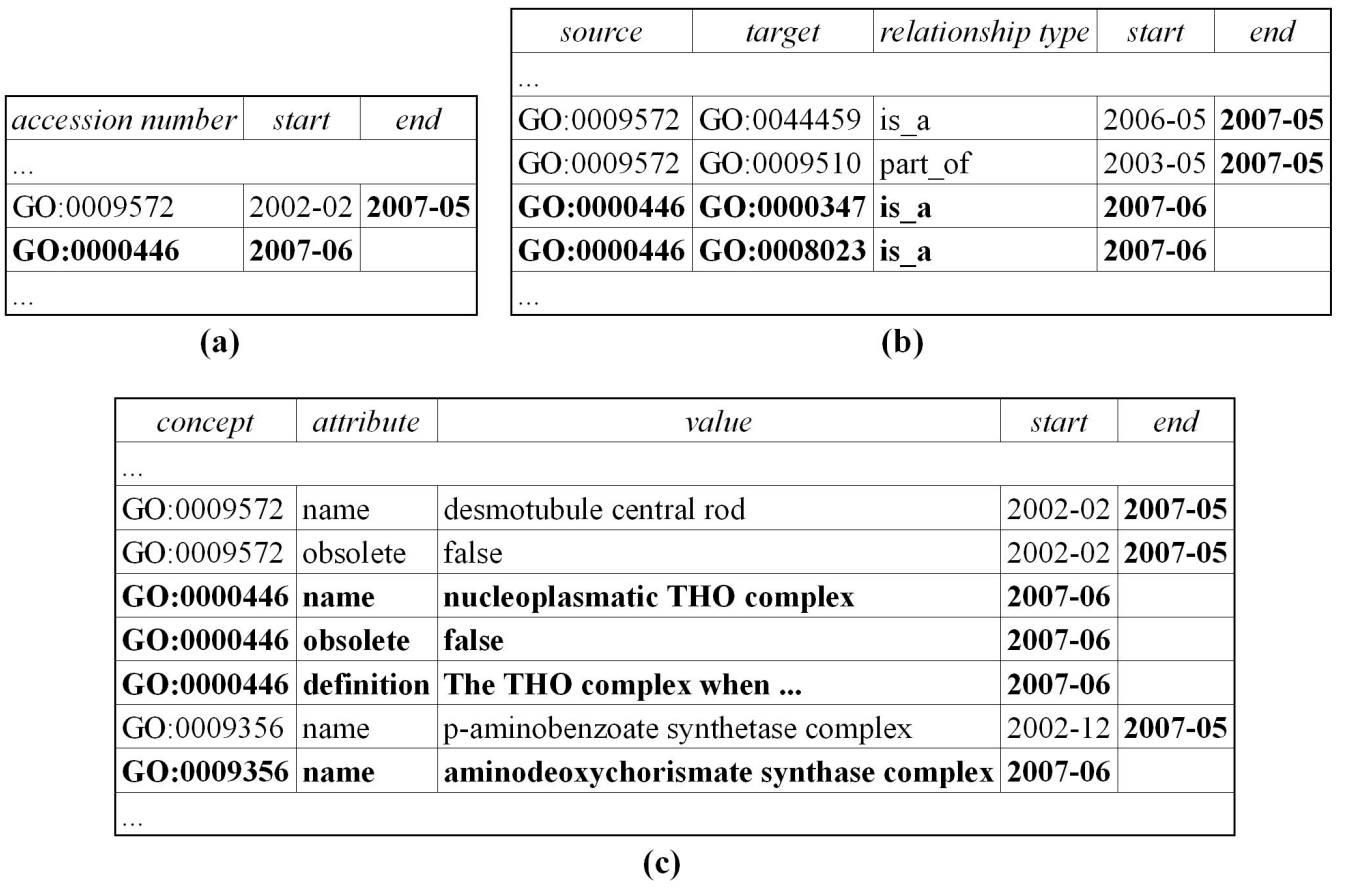
**Extract of the version repository**. Selected modifications on the version repository caused by the evolution of GO cellular components between May and June 2007. (a) concepts, (b) relationships, (c) attributes.

The example in Figure 2 illustrates selected changes that occurred during the version change of GO cellular components between May and June 2007 resulting in adaptations of repository elements (values printed in bold indicate changes). GO:0009572 – *desmotuble central rod *(introduced in December 2002) has been deleted. Thus, the end timestamps of this concept as well as its attributes and relationships are set to 2007-05. In contrast, the concept GO:0000446 – *nucleoplasmic THO complex *with attributes (obsolete = false and a definition string) and relationships (GO:0000446-GO:0000347, GO:0000446-GO:0008023) was added to the ontology. As a result the system creates new entries with a start timestamp of 2007-06. The name of GO:0009356 has changed from *p-aminobenzoate synthetase complex *to *aminodeoxychorismate synthase complex*, hence the end timestamp of the corresponding attribute entry is set to 2007-05 and a new entry with start timestamp 2007-06 is inserted.

The repository is updated periodically with automatic routines which load and integrate the latest ontology versions from the distributor sites. While only few ontology distributors (for example the Gene Ontology consortium) release versions every day we currently consider at most one version per month (in case of several versions we pick the first one). Of course, all changes that occurred between two releases are captured by comparing the monthly ontology versions. The repository is not limited to a particular ontology format such as OBO, but we can customize the import to integrate ontologies of various formats (for example CSV, OWL, relational databases).

### Web application

**OnEX **allows the exploration of ontology evolution by providing three interactive workflows: (1) the general Quantitative evolution analysis, (2) the Concept-based analysis and (3) the Annotation migration workflow. In the following these workflows are explained in detail.

#### Quantitative analysis

The quantitative evolution analysis provides an overview about the evolution of the ontologies supported by **OnEX**. An overview table comparatively summarizes change statistics and the development of the ontology sizes. For a selected ontology, trend charts illustrate the evolution history. Different tables display the changes that occurred between ontology versions, in particular added, marked obsolete, fused and deleted concepts. Users can thus first compare the overall development of different ontologies and then focus on a specific ontology of interest. They can identify phases of ontology stability or instability to estimate the potential impact of ontology changes on annotations and analysis results of interest. Moreover, they can quickly find new or changed ontology concepts.

**Figure 3:**
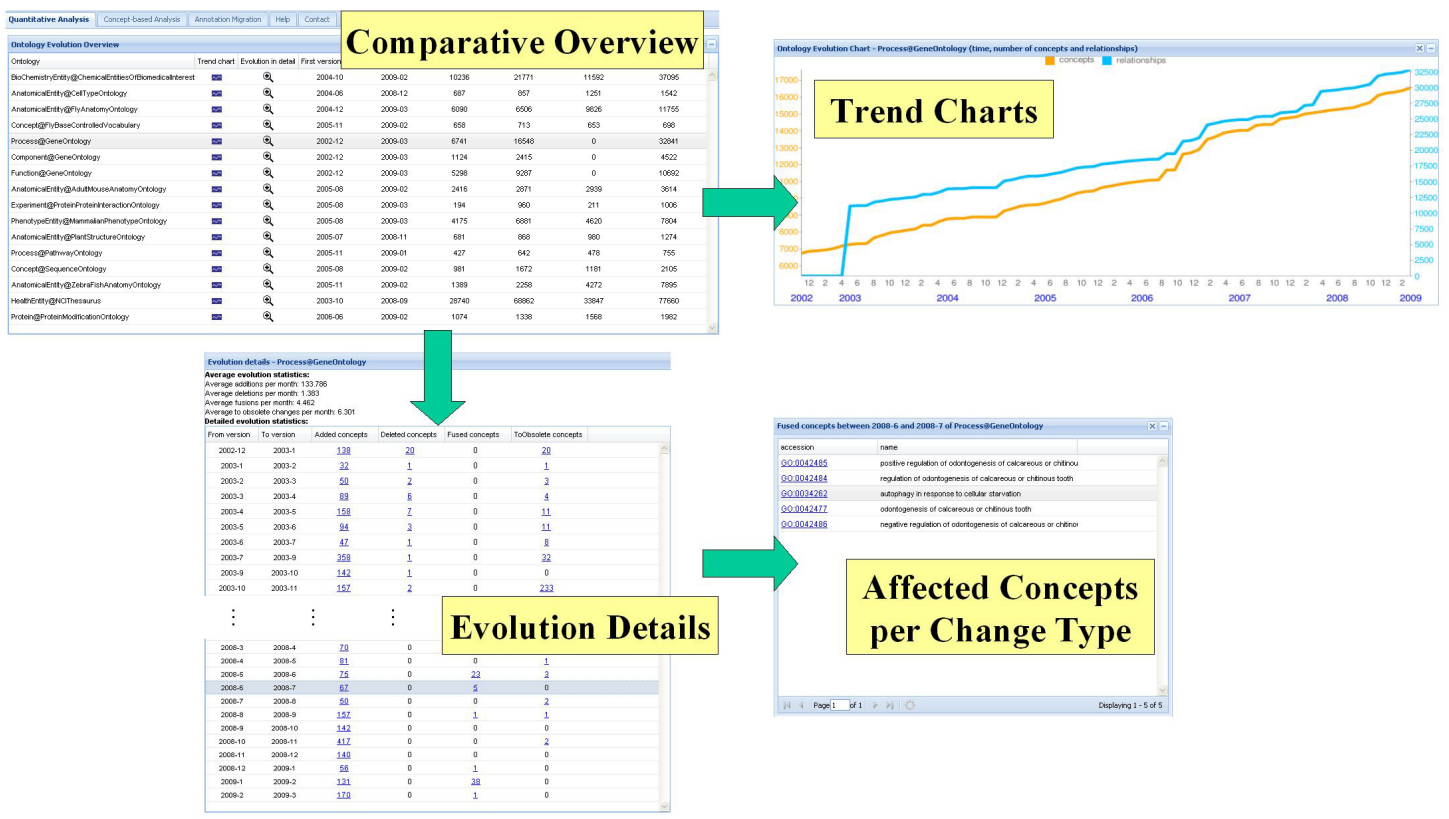
**Quantitative analysis workflow**. Parts of the quantitative analysis workflow.

An application of the first workflow is illustrated in the example scenario of Figure 3 focusing on changes in the GO sub-ontology biological processes (BP). The overview panel *(Comparative Overview) *shows basic statistics of all available ontologies. For instance, it is indicated that GO BP consists of approx. 16,500 concepts interconnected by 33,000 relationships in the March 2009 version as opposed to only 7,000 concepts and no relationships in the first available version of December 2002. The *Trend Chart *for GO BP illustrates a steady increase in the number of both concepts and relationships. It can be observed that relationships have been introduced in April 2003 and that a significant increase occurred between July 2006 and December 2006. Users can then navigate to *Evolution Details *to see average evolution statistics and quantitative changes between the captured ontology versions. For instance, the GO BP sub-ontology experienced approx. 130 concept additions per month while on average about 12 concepts are modified per month, i.e., have become obsolete or were deleted. The exact number of added, deleted, fused and obsolete marked concepts is displayed in a table that can be sorted according to different criteria such as the number of affected concepts. One can see that most additions occurred between the versions of September and November 2006 (971 concepts). If a user is interested in a specific version change, she may navigate to a further panel displaying the *Affected Concepts per Change Type*. As an example, in the July 2008 version of GO BP five concepts have become obsolete, e.g., GO:0034262 (*autophagy in response to cellular starvation*) and GO:0042477 (*odontogenesis of calcareous or chitinous tooth*). As a next step the user may navigate to details about the affected concepts by clicking on the accession number (see workflow in the following section).

#### Concept-based analysis

The concept-based evolution analysis workflow supports a detailed change analysis for specific concepts of interest. This workflow should also be relevant to curators of annotations to inspect the history of a specific concept to notice former changes such as re-definitions or new synonyms which may be useful for the manual annotation process. The workflow starts with a search for the relevant concepts, either by accession number or a keyword search across all or selected ontologies. The workflow can also be accessed from the quantitative evolution analysis workflow by selecting a concept of interest. The system provides current information about the selected concepts as well as the concept history in tabular form.

**Figure 4:**
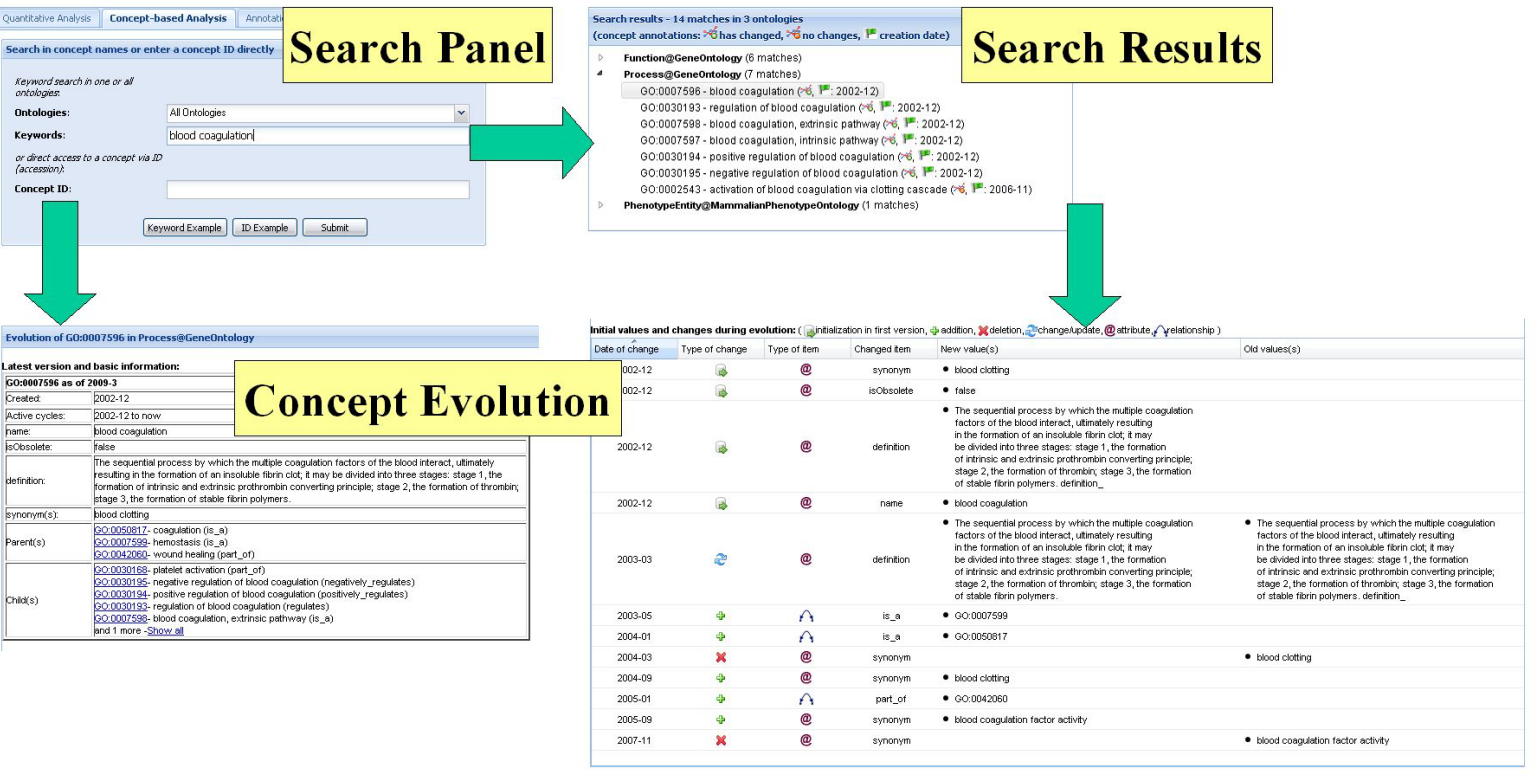
**Concept-based analysis workflow**. Parts of the concept-based analysis workflow.

The scenario in Figure 4 illustrates the concept-based evolution analysis for blood coagulation concepts across all ontologies. We first use a string-based keyword search for the expression *blood coagulation *(*Search Panel*) delivering *Search Results*, i.e., matching terms in the selected ontologies. In the example we obtain 7 matches for the GO BP sub-ontology and further matches in two other ontologies. The user may now select a matched term of interest to inspect its history in more detail (here GO:0007596 – *blood coagulation *of GO BP). The resulting *Concept Evolution *panel has two parts. The first part provides information about the concept in the latest version (name, accession number, definition, synonyms, obsolete status, parents and children) and some historical statistics (creation date or periods of non-existence). The second part presents the history of the concept in tabular form. The table indicates the initial status (attribute values, relationships) of a concept at creation time and lists all concept changes such as additions, modifications or deletions of attribute values and relationships. For instance, the *blood coagulation *concept has been introduced in December 2002 and was available in all versions until now. Parent relationships to GO:0007599 (*hemostasis*), GO:0050817 (*coagulation*) and GO:0042060 (*wound healing*) were added between 2003 and 2005. Other changes affected the synonyms, for example the expression *blood clotting *was temporarily deleted and *blood coagulation factor activity *was only present between 2005 and 2007.

#### Annotation migration component

Finally, **OnEX **allows the migration of annotations after the release of a new ontology version. Out-dated annotations can be detected and automatically migrated to a newer (usually the latest) ontology version. The updated annotations may then be used to rerun an analysis that was originally performed for older ontology versions. The annotations to be updated are provided by an annotation file. The migration component accepts CSV annotation formats such as the GOA format. The migration workflow is based on the analysis of differences between the ontology version utilized in the user annotation file and the new ontology version. The adaptation of the annotations relies on the detected change types.

**Figure 5:**
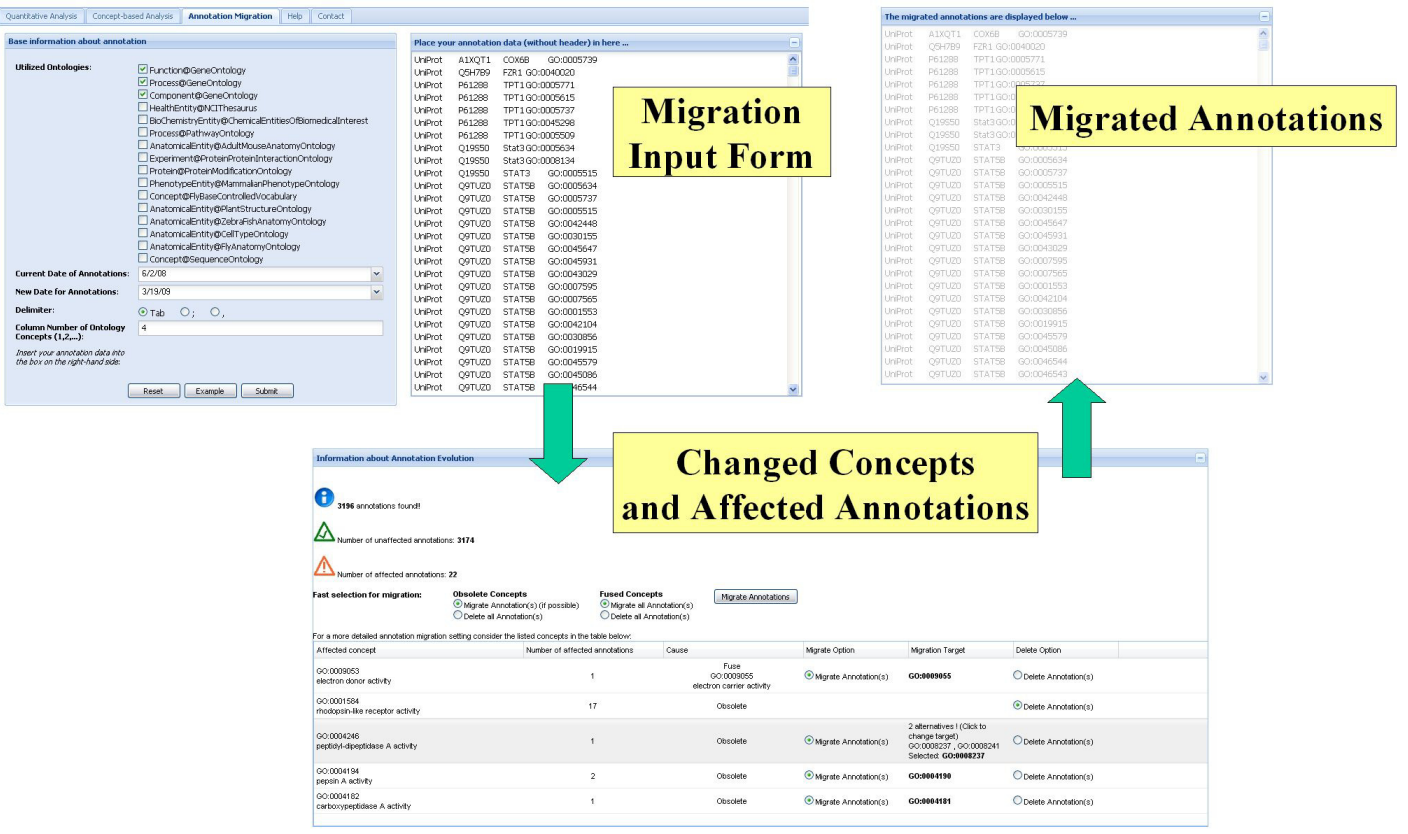
**Annotation migration workflow**. Parts of the annotation migration workflow.

The example in Figure 5 uses a data set with pig gene product associations from AgBase [12] using the Gene Ontology version of May 2008; the annotations should be migrated to the current GO version. The *Migration Input Form *allows users to specify the parameters for migrating their annotations, in particular the date of the ontologies utilized in the annotations, the new date of ontologies, information about the format and the annotations themselves (which may be simply copied into a text area).

After submitting the data to the system, the *Changed Concepts and Affected Annotations *are reported based on the computation of changes between the relevant ontology versions. We focus on three change types that affect the annotations: deleted concepts, fused concepts and concepts marked as obsolete. A result table displays the observed ontology changes and the number of affected annotations. Now the user has different possibilities to migrate the out-dated annotations to the new ontology version. In case of deletions the corresponding annotations are deleted as well. Annotations to obsolete concepts can be deleted or updated. As some ontology distributors provide links to alternative concepts after setting them to obsolete, an annotation deletion can often be avoided. We thus integrated mappings between obsolete and alternative concepts in our repository. For instance, GO offers such mappings on their website  and some OBO ontologies specify alternatives within term descriptions or comments. In case of multiple migration alternatives the user can decide to which ontology concept affected annotations should be migrated. For concepts to be fused to another concept one may migrate the annotations to the fused concept or delete the annotations. Users can set migration options for a complete change type or can set individual specifications. The example of the pig gene product annotations of May 2008 possesses one fused and four obsolete ontology concepts resulting in 22 affected annotations. For instance, the discovered obsolete concept GO:0004246 (*peptidyl-dipeptidase A activity*) has two migration alternatives (GO:0008237 – *metallopeptidase activity*, GO:0008241 – *peptidyl-dipeptidase activity*) between which the user can choose. Finally, the specified migration settings are applied to migrate the out-dated annotations to the new ontology version. The *Migrated Annotations *results are provided in an output panel and can be downloaded for further processing.

## Results

As of April 2009 **OnEX **integrates about 560 versions of 16 life science ontologies from different ontology sources, such as Gene Ontology and OBO Foundry dating back to 2002. The version management presented in the implementation section supports an efficient storage of a large amount of ontology versions. Currently, the repository includes approx. 150,000 concepts, 260,000 relationship and 1,000,000 attribute entries which occur in different ontology versions. To avoid duplicate storage of unchanged ontology parts we only store differences between ontology versions after the first version. A specific ontology version can be retrieved by merely providing the timestamp. **OnEX **is not limited to a particular ontology format and can thus flexibly include further ontologies in the future.

The web application contains help pages describing the workflows step-by-step for novice users. Sample data is provided to follow the workflows and see how the system works. **OnEX **is running since June 2008 and has been tested by five different life science research groups at the University of Leipzig. Moreover, the system is also utilized within the German-wide MediGRID community [39].

## Conclusion

**OnEX **provides an interactive and user-friendly access to valuable information about evolutionary changes in life science ontologies. Users are able to detect changes that occurred in an ontology version they utilize or plan to apply. For instance, one can estimate how intensive the analysis results are affected by ontology evolution and how frequent rerunning an analysis may be advised. **OnEX **is available at  with currently approx. 560 versions of 16 life science ontologies.

Three workflows of the system address different analysis aspects: the comparative quantitative analysis of different life science ontologies, the detailed inspection of single ontology concept histories and the semi-automatic migration support for out-dated analysis results (especially annotations of molecular biological objects).

For future work, we plan to extend the system by additional change types and the identification of (un)stable ontology regions. We further plan to establish a "traffic-light" visualization for ontologies to display their stability or instability in an intuitive manner. Finally, the three-tier architecture can be extended by a web service interface to allow a software-based access to the stored ontology versions as well as the change analysis routines.

## Availability and requirements

• **Project name**: OnEX – Ontology Evolution Explorer

• **Project home page**: 

• **Operating system(s)**: Platform independent

• **Programming language**: Java

• **Any restrictions to use by non-academics**: none

## Authors' contributions

MH has designed and implemented the web application. The middleware and the data server including ontology versioning, import functionalities and further components were realized by MH and TK. AG participated in the GUI component design, the description of the help pages as well as testing the system. ER provided higher-level supervision and coordinated the project. All authors contributed to, read and approved the final manuscript.
